# Using Crash Outcome Data Evaluation System (CODES) to examine injury in front vs. rear-seated infants and children involved in a motor vehicle crash in New York State

**DOI:** 10.1186/s40621-021-00328-8

**Published:** 2021-06-21

**Authors:** Michael Bauer, Leah Hines, Emilia Pawlowski, Jin Luo, Anne Scott, Matthew Garnett, Morgan Uriell, Joyce C. Pressley

**Affiliations:** 1 New York State Department of Health, Bureau of Occupational Health & Injury Prevention, Center for Environmental Health, Corning Tower, Room 1325, Empire State Plaza, Albany, NY 12237 USA; 2 Department of Epidemiology, Mailman School of Public Health, Columbia University, New York, NY 10032 USA; 3 Departments of Epidemiology and Health Policy and Management and the Center for Injury Epidemiology and Prevention at Columbia, Mailman School of Public Health, Columbia University, New York, NY 10032 USA; 4 Columbia Center for Injury Science and Prevention, Mailman School of Public Health, Columbia University, New York, NY 10032 USA

**Keywords:** Motor vehicle injury, Children, Seating position, Child safety seats, Restraint use

## Abstract

**Background:**

In New York State (NYS), motor vehicle (MV) injury to child passengers is a leading cause of hospitalization and emergency department (ED) visits in children aged 0–12 years. NYS laws require appropriate child restraints for ages 0–7 years and safety belts for ages 8 and up while traveling in a private passenger vehicle, but do not specify a seating position.

**Methods:**

Factors associated with injury in front-seated (*n* = 11,212) compared to rear-seated (*n* = 93,092) passengers aged 0–12 years were examined by age groups 0–3, 4–7 and 8–12 years using the 2012–2014 NYS Crash Outcome Data Evaluation System (CODES). CODES consists of Department of Motor Vehicle (DMV) crash reports linked to ED visits and hospitalizations. The front seat was row 1 and the rear rows 2–3. Vehicle towed from scene and air bag deployed were proxies for crash severity. Injury was dichotomized based on Maximum Abbreviated Injury Severity (MAIS) scores greater than zero. Multivariable logistic regression (odds ratios (OR) with 95% CI) was used to examine factors predictive of injury for the total population and for each age group.

**Results:**

Front-seated children had more frequent injury than those rear-seated (8.46% vs. 4.92%, *p* < 0.0001). Children in child restraints experienced fewer medically-treated injuries compared to seat belted or unrestrained children (3.80, 6.50 and 13.62%, *p* < 0.0001 respectively). A higher proportion of children traveling with an unrestrained vs. restrained driver experienced injury (14.50% vs 5.26%, *p* < 0.0001). After controlling for crash severity, multivariable adjusted predictors of injury for children aged 0–12 years included riding in the front seat (1.20, 1.10–1.31), being unrestrained vs. child restraint (2.13, 1.73–2.62), being restrained in a seat belt vs. child restraint (1.20, 1.11–1.31), and traveling in a car vs. other vehicle type (1.21, 1.14–1.28). Similarly, protective factors included traveling with a restrained driver (0.61, 0.50–0.75), a driver aged < 25 years (0.91, 0.82–0.99), being an occupant of a later vehicle model year 2005–2008 (0.68, 0.53–0.89) or 2009–2015 (0.55, 0.42–0.71) compared to older model years (1970–1993).

**Conclusions:**

Compared to front-seated children, rear-seated children and children in age-appropriate restraints had lower adjusted odds of medically-treated injury.

## Introduction

In New York State (NYS), motor vehicle (MV) injury to child passengers is a leading cause of hospitalization and emergency department (ED) visits in children aged 0–12 years (National Highway Traffic Safety Association, 2008; CDCs Web injury Query, n.d.; New York State Department of Health, Bureau of Occupational Health and Injury Prevention, n.d.). Seat belts are generally a very effective safety device for protecting MV occupants, but seat belts alone do not properly fit a child’s small body and do not offer the same protection they afford adult occupants (Eby et al., 2005). Infant and child restraint guidelines from the National Highway Traffic Safety Administration (NHTSA) and American Academy of Pediatrics (AAP) recommend rear-facing restraints for infants, forward facing child restraints when rear-facing restraints have been outgrown, and use of belt-positioning booster seats for children that are at least 4 years of age, 40 pounds, no longer fit forwarding facing child safety seats, but are still too young (less than 8 years of age) or too small (less than 57 in.) to fit properly into seat belts alone (NHTSA, n.d.-a; Durbin & American Academy of Pediatrics, 2011; Huang et al., 2019; Sartin et al., 2020; Durbin et al., 2018). NHSTA also recommends that all children under age 13 years ride in the rear seat (NHTSA, n.d.-a).

Though most states and the District of Columbia have laws requiring appropriate child restraints, these laws vary according to age, height, and weight across states. The NYS child restraint law requires children aged 0–7 years to be restrained in an appropriate child restraint system, and children ages 8 and up to use a seat belt. These laws do not specify front- vs. rear-seating position (The New York State Governor’s Traffic Safety Committee, n.d.; GHSA, n.d.; IIHS/HLDI, n.d.). NYS child restraint laws have been shown to be an effective way at reducing MV injuries in children (Sun et al., 2010). Previous research comparing child restraints and seat belts alone for children aged 2–6 years has demonstrated that child restraint systems offer considerable safety advantage over seat belts (Macy et al., 2014; Smola et al., 2020). Child restraints are specially designed to help reduce the risk of ejection during a crash as well as to better distribute the force of the crash through stronger bones rather than soft tissues (Durbin et al., 2003; Elliott et al., 2006; Winston et al., 2007). Since the addition of front seat passenger airbags, where a child sits in a MV has become increasingly important to safety (Ferguson et al., 2000). Previous studies have shown that the rear seating position offers substantial protection (Petridou et al., 1998; Berg et al., 2000). A study conducted in 2006 by Smith and Cummings found that the risk of death was 21% lower among passengers in the rear seat compared with front-seated passengers, with the rear seat offering the most protection for children 0–12 years old (Smith & Cummings, 2006). However, more recent discussion has centered around improved vehicle design and whether the front seat has become less dangerous for young front-seated occupants.

Several recent changes in vehicle design, vehicle safety features and infant and child safety seat design underscore the need for further examination of MV crash outcomes involving a child passenger. This paper provides an update of the relationship between rear-vs. front-seating positions, restraint use and injury in NYS children aged 0–12 years and by age group for children involved in nonfatal as well as fatal MV crashes.

## Methods

### Study population

The study population included children involved in a MV crash while riding as a passenger seated in rows 1–3 of a 4-wheeled passenger vehicle. Vehicles included convertibles, sedans, SUVs, vans and pickup trucks. Passengers on motorcycles or in other vehicle types were excluded as were children riding in the cargo area or hanging onto/outside the vehicle. Trend analyses were performed using the NYS Crash Outcome Data Evaluation System (CODES) data from the years 2003–2014. A subpopulation analysis of predictors of child injury used CODES 2012–2014. In 2014, NYS had 3,018,072 children aged 0–12 years.

### Data source(s)

CODES was used in this study of child occupants involved in a MV crash on a NYS roadway (NHTSA, n.d.-b). CODES is a database made by merging multiple data sources using probabilistic linkage methods. It is created by matching individual records from the NYS DMV Accident Information System (AIS) to the NYS Department of Health (DOH) Statewide Planning and Research Cooperative System (SPARCS) database of ED visits and hospitalizations. The probabilistic linkage was performed using LinkSolv Software (Strategic Matching, Inc., Morrisonville, New York). The all age CODES database linkage is robust, with strong and consistent linkage probabilities, and a high sensitivity for accurately linking cases. For linkage, cases require both a pre-hospital MV crash report as well as an inpatient or outpatient record.

The work had IRB approval through IRBNet protocol number 14–044.

### Variable definitions: outcome, exposure and covariates

#### Outcomes

##### Injury

The dependent variable was defined as medically-diagnosed injury for child occupants aged 0–12 years involved in MV crashes. Injury was dichotomized using categories of the Maximum Abbreviated Injury Score (MAIS). The MAIS is calculated using ICD-9-CM diagnosis codes of hospital-treated patients. Injury was then dichotomized as either injured or not injured. Any MAIS score greater than 0 was defined as injured, and any score equal to 0 was defined as uninjured (Ferreira et al., 2017)*.*

#### Child passenger and driver characteristics

##### Child seating position

Seating position was dichotomized as rear- vs. front-seated. Children in row 1 (the driver’s seat, front middle, and front right position) were defined as front-seated. Children seated in rows higher than row 1 were defined as being rear-seated.

##### Child age group

The age groups of children were categorized as 0–3 years, 4–7 years, and 8–12 years of age.

##### Child gender

The gender of the child was defined as male or female.

##### Child safety equipment

Multiple variables for child safety equipment were categorized as: 1) restrained in a child restraint; 2) restrained in a seat belt only; or 3) unrestrained. Seat belt included lap belt, harness, lap belt/harness, air bag deployed/lap belt, air bag deployed/harness, or air bag deployed/lap belt/harness. Child restraint was defined as child restraint only and air bag deployed/child restraint. Unrestrained was defined as no restraint used or airbag deployed without a restraint.

##### Driver’s age

The age of the driver at the time of the crash was dichotomized as less than 25 years of age or 25 years and older. These age groupings were selected to control for smaller frequencies of younger drivers in our sample. Additionally, drivers under the age of 25 are more likely to be involved in crashes and engage in riskier driving behaviors than their more experienced counterparts (Centers for Disease Control and Prevention, n.d.)*.*

##### Driver safety equipment

The driver’s safety equipment was dichotomized as restrained or unrestrained. Restrained was defined as lap belt, harness, lap belt/harness, air bag deployed/lap belt, air bag deployed/harness, and air bag deployed/lap belt/harness. Unrestrained was defined as no restraint used and included airbag deployed without a restraint.

#### Vehicle and crash characteristics

##### Vehicle year

Vehicle model year was categorized as 1970–1993, 1994–1997, 1998–2004, 2005–2008, or 2009 and newer (Ryb et al., 2011)*.* Older vehicles were categorized as 1997 or older and Newer vehicles as 1998 and newer.

##### Vehicle type

Vehicle body type was limited to 4-wheeled passenger vehicles and defined as two categories: 1) cars and 2) other 4-wheeled passenger vehicles including light trucks, suburbans, sport utility vehicles (SUVs) and vans.

##### Crash severity

Vehicle airbag deployed and vehicle towed from the scene were used as proxies for crash severity. Both variables were dichotomous. Airbag deployment at the time of the crash was coded yes or no for both restrained and unrestrained child passengers.

#### Compliance with safety recommendations

Injury in children being transported in compliance with safety recommendations from the NHTSA and with applicable NYS laws were evaluated where data were available. We were able to ascertain rear- vs. front-seating position, but not the forward or backward direction the child restraint was facing. NYS laws require children ages 0–7 years to be restrained in the appropriate child restraint system and that children aged 8 and up years use a seat belt. These laws do not specify a seating position (The New York State Governor’s Traffic Safety Committee, n.d.; GHSA, n.d.)*.*

### Statistical analysis

Bivariate associations of demographic, behavioral, vehicle and crash characteristics were assessed using chi-square tests and logistic regression. Multivariable backward stepwise logistic regression analyses were performed to evaluate the adjusted association between seating position (front seat vs. rear seat) and the outcome in the 2012–2014 NYS CODES linked data. The outcome variable, medically-diagnosed injury for child occupants aged 0–12 years involved in a MV crash was dichotomized using the MAIS calculated from ICD-9-CM diagnosis codes (Ferreira et al., 2017)*.* MAIS ranged from 0 (uninjured) to 6 (maximum, untreatable) injury. For statistical analyses using backward stepwise logistic regression, the outcome was dichotomized as uninjured (MAIS = 0) or injured (fatality or MAIS of 1–6). Independent variables assessed in the models included child seating position (front seat vs. rear seat), child safety equipment (unrestrained, child restraint or seat belt only), child age group (0–3 years, 4–7 years, or 8–12 years), child gender (male vs. female), vehicle year (1970–1993, 1994–1997, 1998–2004, 2005–2008, > = 2009), vehicle type (car vs. other vehicles), airbag deployed (yes or no), vehicle towed from scene (yes or no), driver safety equipment (restrained vs. unrestrained) and driver age (under 25 years vs 25 years and over). Analyses were performed examining airbag deployment and injury in front and rear-seated children for vehicle model years 1994–1997 vs. 1998–2015. SAS Proc Mianalyze was used for multiple data imputation and logistic regression was used for multivariable analyses. Statistical significance was defined as p < 0.05. Odds ratios (OR) are provided with 95% confidence intervals (CI). Analyses were performed using SAS 9.4 (SAS Institute Inc., 2014)*.*

## Results

NY state population consists of just over 3 million children aged 0–12 years. During the 3 year timeframe of this study, 104,304 of NYS’s children aged 12 years and under were involved in a MV crash (Table 1). Of these − 93,092 (89.3%) were rear-seated and 11,212 (10.7%) were front-seated.

**Table 1:** Population characteristics for pediatric occupants aged 0–12 years with and without injury

	All Ages Bivariate Analysis *n (%)*		
	Injury	No Injury	***p***-value
Gender^a^			< 0.0001
Female	2953 (5.66)	49,237 (94.34)	
Male	2572 (4.95)	49,379 (95.05)	
Age group			< 0.0001
0–3	1078 (3.17)	32,920 (96.83)	
4–7	1702 (5.21)	30,948 (94.79)	
8–12	2749 (7.30)	34,907 (92.70)	
Seating Position			< 0.0001
Front	948 (8.46)	10,264 (91.54)	
Rear	4581 (4.92)	88,511 (95.08)	
Child safety equipment^b^			< 0.0001
Child Restraint	1832 (3.80)	46,403 (96.02)	
Seatbelt	3321 (6.50)	47,799 (93.50)	
Unrestrained	185 (13.62)	1173 (86.38)	
Driver Safety Equipment^c^			< 0.0001
Restrained	5117 (5.26)	92,168(94.74)	
Unrestrained	162 (14.50)	955(85.50)	
Airbag Deployed^d^			< 0.0001
Deployed	428 (20.83)	1627 (79.17)	
Not Deployed	4915 (4.98)	93,804 (95.02)	
Vehicle Towed			< 0.0001
Towed	3760 (11.53))	28,845 (88.47)	
Not Towed	1769 (2.47)	69,930 (97.53)	
Vehicle Type			< 0.0001
Passenger Car	2770 (6.31)	41,157 (93.69)	
Other Vehicle	2759 (4.57)	57,618 (95.43)	
Vehicle Year^e^			< 0.0001
1970–1993	74 (7.86)	868 (92.14)	
1994–1997	381 (9.19)	3764 (90.81)	
1998–2004	2074 (6.69)	28,639 (93.31)	
2005–2008	1408 (5.07)	26,378 (94.93)	
2009 and Newer	1389 (3.87)	34,547 (96.13)	
Driver Age^f^			< 0.0001
Under 25	606 (6.30)	9011 (93.70)	
25 or Greater	4885 (5.23)	88,459 (94.77)	

^a^Gender missing (*n* = 163); ^b^Child Safety Equipment missing (*n* = 3591); ^c^Driver Safety Equipment missing (*n* = 5902); ^d^Airbag Deployed (*n* = 3530); ^e^Vehicle Year (*n* = 4810); ^f^Driver’s Age (*n* = 1343)

### Unadjusted and adjusted multivariable predictors of injury for front- vs. rear-seated 0–12 year olds

**Fig. 1:**
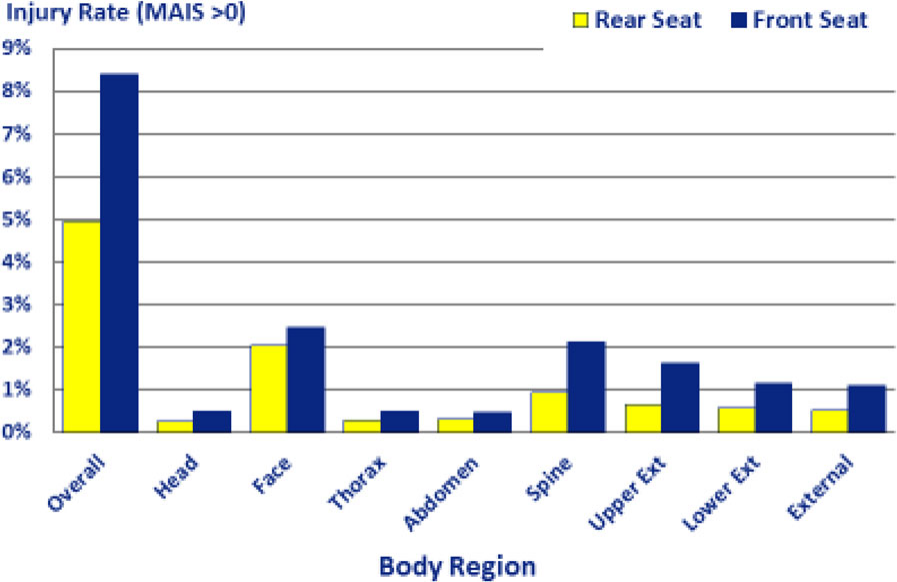
Distribution of injuries by Body Region Children Aged 0-12 years in Motor Vehicle Crashes, NYS, 2012–2014

Rear seat use among child occupants aged 0–12 years increased during the study timeframe. Compared to front-seated children, those rear-seated had lower medically diagnosed injury as indicated by ED visits and hospitalizations. Rear-seated children had lower observed medically diagnosed injury (MAIS > 0) in 8 of all 9 body regions, as compared to children in the front seat (Fig. 1).

After adjustment for other variables associated with injury (such as child restraint use, age, gender, vehicle type, vehicle year, crash severity (as measured by airbag deployed or vehicle towed), driver’s age and driver’s restraint use, the effect of being a front-seated child aged 0–12 years remained significantly higher than child passengers who were rear-seated (Table 2). The strongest predictor of adjusted injury in passengers aged 0 to 12 years was crash severity as measured by airbag deployment and vehicle towed from the scene. Children aged 0 to 12 years were more than twice as likely to be injured if the airbag deployed and nearly 5 times more likely to be injured if the vehicle had to be towed (Table 2).

**Table 2:** Predictors of injury among child passengers ages 0–12: Unadjusted and Adjusted Odds Ratios, Crash Outcome Data Evaluation System (CODES), 2012–2014

Parameter	Unadjusted		Multivariable Adjusted	
	OR	95% CI	OR	95% CI
Child seating position (Front seat vs. Rear seat)	1.78	(1.66–1.92)	1.20	(1.10–1.31)
Child safety equipment (Unrestrained vs. Child restraint)	3.00	(2.10–4.27)	2.13	(1.73–2.62)
Child safety equipment (Seat belt vs. Child restraint)	1.50	(1.22–1.83)	1.20	(1.11–1.31)
Child gender (Female vs. Male)	1.20	(1.10–1.32)	1.14	(1.08–1.21)
Airbag deployed (Yes vs. No)	2.48	(2.10–2.97)	2.36	(2.10–2.67)
Vehicle year (1994–1997 vs. 1970–1993)	1.02	(0.69–1.52)	1.17	(0.89–1.55)
Vehicle year (1998–2004 vs. 1970–1993)	0.73	(0.50–1.06)	0.85	(0.66–1.10)
Vehicle year (2005–2008 vs. 1970–1993)	0.56	(0.39–0.82)	0.68	(0.53–0.89)
Vehicle year (2009 and greater vs. 1970–1993)	0.47	(0.31–0.68)	0.55	(0.42–0.71)
Vehicle type (Car vs. Other vehicles)	1.21	(1.11–1.32)	1.21	(1.14–1.28)
Vehicle towed from scene (Yes vs. No)	4.18	(3.82–4.58)	4.91	(4.61–5.23)
Driver safety equipment (Restrained vs. Unrestrained)	0.99	(0.69–1.42)	0.61	(0.50–0.75)
Driver’s age < 25 years (Yes vs. No)	1.03	(0.87–1.22)	0.91	(0.82–0.99)

### Multivariable predictors of injury for front- vs. rear-seated 0–3 year olds

Predictors of injury in the youngest age group are shown in Table 3. Measures of crash severity were highly predictive of incurring a medically-treated injury in 0–3 year olds. For very young children, vehicle towed from the scene was associated with a nearly 7 fold higher odds of injury, with airbag deployment showing nearly twice the odds of injury (Table 3). Driver use of safety equipment was protective against young child injury (Table 3).

**Table 3:** Predictors of injury among child passengers ages 0–3,4–7, and 8–12: Unadjusted and Adjusted Odds Ratios, Crash Outcome Data Evaluation System (CODES), 2012–2014

Parameter	Unadjusted	Multivariable Adjusted	Unadjusted	Multivariable Adjusted	Unadjusted	Multivariable Adjusted
	Ages 0–3 OR (95% CI)	Ages 0–3 OR (95% CI)	Ages 4–7 OR (95% CI)	Ages 4–7 OR (95% CI)	Ages 8–12 OR (95% CI)	Ages 8–12 OR (95% CI)
Child seating position (Front seat vs. Rear seat)	1.26 (1.03–1.53)		1.41 (1.25–1.59)	1.16 (1.03–1.31)	1.36 (1.30–1.43)	1.16 (1.05–1.28)
Child safety equipment (Unrestrained vs. Child restraint)	2.14 (1.48–3.10)		2.57 (1.96–3.37)	1.68 (1.19–2.36)	4.03 (3.06–5.32)	3.12 (2.29–4.25)
Child safety equipment (Seat belt vs. Child restraint)	0.98 (0.82–1.17)		1.20 (1.08–1.32)	1.22 (1.09–1.35)	1.55 (1.29–1.88)	1.50 (1.22–1.84)
Child gender (Female vs. Male)	0.93 (0.86–0.99)		1.16 (1.10–1.23)		1.19 (1.14–1.24)	1.20 (1.10–1.30)
Airbag deployed (Yes vs. No)	4.23 (3.65–4.90)	1.91 (1.51–2.59)	4.56 (4.01–5.17)	2.31 (1.82–2.94)	5.00 (4.59–5.46)	2.52 (2.14–2.98)
Vehicle year (1994–1997 vs. 1970–1993)	1.93 (1.02–3.66)		1.14 (0.70–1.44)		0.99 (0.69–1.40)	0.99 (0.68–1.44)
Vehicle year (1998–2004 vs. 1970–1993)	1.26 (0.69–2.32)		0.91 (0.57–1.44)		0.65 (0.47–0.90)	0.70 (0.50–0.99)
Vehicle year (2005–2008 vs. 1970–1993)	0.98 (0.53–1.81)		0.67 (0.42–1.06)		0.49 (0.35–0.68)	0.54 (0.38–0.77)
Vehicle year (2009 and greater vs. 1970–1993)	0.63 (0.34–1.16)		0.55 (0.35–0.89)		0.37 (0.27–0.52)	0.44 (0.31–0.63)
Vehicle type (Car vs. Other Vehicles)	0.75 (0.70–0.81)		0.64 (0.61–0.68)	1.29 (1.16–1.44)	0.70 (0.67–0.73)	1.21 (1.11–1.32)
Vehicle towed from scene (Yes vs. No)	7.08 (6.52–7.68)	6.9 (5.87–8.09)	5.45 (5.13–5.79)	4.99 (4.46–5.58)	4.53 (4.33–4.75)	4.20 (3.85–4.59)
Driver safety equipment (Restrained vs. Unrestrained)	0.32 (0.27–0.39)	0.43 (0.33–0.70)	0.29 (0.24–0.33)	0.48 (0.34–0.66)	0.42 (0.36–0.49)	
Driver’s age < 25 years (Yes vs. No)	1.46 (1.34–1.59)		1.58 (1.43–1.73)	0.83 (0.69–0.99)	1.39 (1.28–1.51)	

### Multivariable predictors of injury for front- vs. rear-seated 4–7 year olds

In comparison to 4–7 year olds who were rear-seated, front-seated children in this age group were 24% more likely to be injured after controlling for restraint use, child restraint, vehicle type, driver’s age and use of driver restraint, and crash severity (Table 3). Again, measures of crash severity had the highest odds of injury with airbag deployment being associated with more than double the odds of injury and having a vehicle that was towed from the scene an increased odds approximately 5 times the odds compared to children who were in vehicles that were not towed (Table 3). Similar to children aged 0 to 3 years, having a restrained driver was protective against of injury. Having a driver aged less than 25 years of age was protective with children aged 4 to 7 years. Passenger cars showed 29% higher odds of injury compared to other vehicles (Table 3).

### Multivariable predictors of injury for front- vs. rear-seated 8–12 year olds

Front-seated older children aged 8 to 12 years of age were 16% more likely to be injured after controlling for other factors as shown in Table 3. Being unrestrained carried odds of injury that were over 3 times that of restrained children. Even for older children, being in a child restraint was protective. Children in a seat belt compared to a child restraint were 50% more likely to be injured. Airbag deployment was associated with approximately 2.5 times the odds of injury and if the vehicle was towed from the scene, more than 4 times the odds of injury. Passenger cars showed 21% higher odds of injury compared to other vehicles.

### Trends front- vs. rear-seated children

**Fig. 2:**
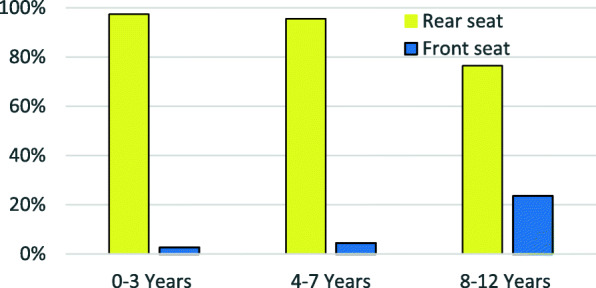
Seating Position by Age Group, 2012–2014

**Fig. 3:**
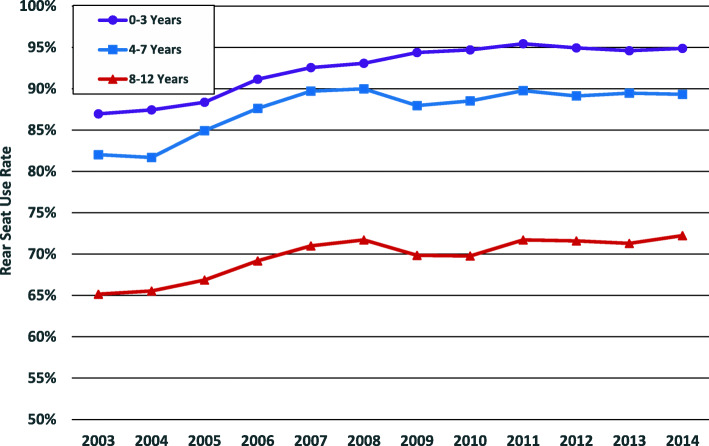
Trends in Rear Seat Use Rate in Children Involved in Motor Vehicle Crashes By Age Group, New York State, 2003-2014

The majority of children in all age groups were rear seated, but this decreased with increasing age across age groups 0–3, 4–7 and 8–12 years (97.4, 95.6 and 76.5%, respectively) (Fig. 2). While significant improvement in compliance with rear seating recommendations have been observed in the state over the last 12 years for all three age groups examined, the relative age disparity has been maintained. Improvements plateaued in 2011 for children aged 7 years and younger while very modest improvement was noted in 8–12 year olds (Fig. 3). Compared to front-seated children, a smaller proportion of rear-seated children aged 0 to 12 years were treated in the ED (11.42% vs. 13.31%) and fewer were admitted to the hospital (0.23% % vs. 0.33%).

The distribution of injury by body region was examined using MAIS. MAIS > 0, was higher in front-seated than in rear-seated children for all body regions except the head. Medically-diagnosed serious injury rates with an MAIS greater than or equal to 4 was higher in front-seated than in rear-seated children (0.74% vs. 0.45%). Traumatic brain injury (TBI) was higher in front-seated than in rear-seated children (1.52% vs. 1.04%).

### Airbag deployment in older vs. newer vehicle models by seating position

Injury in restrained children involved in a MV crash with airbag deployment was higher in older vehicles compared to newer vehicles (33.9% vs. 20.7%). However, this differed in front- vs. rear-seated children. More than half (52.5%) of front-seated children in older vehicles received medically-treated injury compared to 29.7% of rear-seated children. However, this relationship was reversed in rear-seated children where front seated restrained children were less likely to experience medically-treated injury than rear-seated children (4.0% vs. 16.9%).

In crashes of restrained children with no airbag deployment, medically-treated injury was nearly doubled for older compared to newer vehicles (8.7% vs. 4.8%). When front-vs rear-seating position was considered, front-seated children were again significantly more likely to be injured in older vs. newer vehicles (12.1% vs. 7.0%) as were rear-seated children (8.2% vs. 4.5%).

For unrestrained children, airbag deployment was associated with higher injury in older compared to newer vehicles (20.0% vs. 12.2%). In unrestrained children with no airbag deployment, older vehicles were associated with more medically-treated injury (17.4% vs. 11.2%). Rear-seated children were more likely to experience medically-treated injury in older vs. newer vehicles (19.2% vs. 11.3%).

In summary, rear seating of children aged 0–12 years was protective against sustaining a medically-treated injury during a MV crash. Trends in seating positions showed that rear seating of children increased in NYS during the timeframe of this study. After adjusting for other contributing factors, including crash severity, vehicle characteristics, driver characteristics and restraint status and type, riding in the front seat is associated with approximately 20% increase in the odds of having a medically-diagnosed injury in children aged 0–12 years involved in a MV crash. Being front-seated was a significant risk factor for injury across all age groups beyond the 0–3 year age group where there were very few front-seated infants. Children in the rear seat had lower medically diagnosed injury (MAIS > 0) in 8 of all 9 body regions, as compared to children in front seat.

## Discussion

This study used linked hospitalization, ED, and DMV crash report data to examine independent risk and protective factors associated with seating position and injury in children involved in a MV crash. With the exception of our measures of crash severity-- airbag deployment and vehicle towed-- predictors of injury varied across the age categories. Both indicators of crash severity were quite robust and showed a consistent relationship as an adjusted risk factor for MV injury in the total population and in each age group examined. Having a vehicle towed from the scene was the largest indicator of increased childhood injury with the greatest risk observed in the youngest age group, 0–3 years. Having a restrained driver was protective for injury in the younger two age groups, but not the older age group. Older age groups showed adjusted risk ranging from 4 to 5 fold increased in injury, but for the youngest age group the risk of injury for being a passenger in a vehicle that had to be towed from the scene was approximately 7 fold higher, despite the near universal rear seating observed in this age group. Further study is needed to assess forward vs. rear facing restraint use as well as impact locations relative to the seating position of the infants.

Being restrained was significant in unadjusted analyses in all age groups, but was significant in adjusted models only in the two older age groups where both being restrained and restraint type were significant predictors in adjusted models. Our findings are consistent with other studies that have shown that being in a seat belt vs. a child restraint is associated with increased injury in the two older age groups (Durbin et al., 2003; Elliott et al., 2006; Ferguson et al., 2000). An independent protective factor across all age groups included traveling in a newer model vehicle with the more recent the vehicle year, the more protective effect exhibited. Newer model years have been reported previously to be associated with improved crash worthiness (Ryb et al., 2011).

Traveling in the front seat showed a consistent increased adjusted odds of injury of medically-treated injury in all age groups except the youngest 0–3 year olds where the number of front-seated infants was quite small. The older age group was front seated more frequently. This is consistent with previous studies that have documented the increased risk of children being front-seated (Petridou et al., 1998; Berg et al., 2000; Smith & Cummings, 2006; Durbin et al., 2005). Airbag deployment was associated with the highest odds of medically-treated injury in the oldest age group. Although airbag deployment was associated with increased injury risk after controlling for seating position, we could not rule out the possibility that the airbag itself was associated with increased injury in children aged 8–12 years old who were more likely to be front-seated than younger children for whom airbag deployment was also a significant predictor of injury. Driver’s who disregard or devalue the importance of restraint use/restraint laws for themselves may be less likely to properly restraint their children or to see that they self-restrain for older children. Children who self buckle may have no role model or reminding parent driver for buckling.

A vehicle towed from the scene showed especially high odds of injury among children aged 0 to 3 years. In this age group, both towed and airbag deployment were the strongest independent predictors of injury.

There is a lot of variability across states in child restraint laws with regard to age, size, weight and seating positions. Despite guidelines issued by both NHTSA and the AAP that children aged 11 and younger should be rear seated. Fewer than 40% of states explicitly have a stated requirement for children to be rear seated. However, some laws, including NYs which does not include a stated requirement for rear seating, require “appropriate restraint” (NHTSA, n.d.-a; Durbin & American Academy of Pediatrics, 2011). NYS law requires that children be placed in an appropriate child restraint system based on their age until their eighth birthday, when they can legally graduate into using the vehicle’s safety belt (GHSA, n.d.). It is unknown whether the significantly increased injury observed in this study among 8–12 year olds who were restrained by seat belt is due to insufficient height and weight for optimal safety belt fit. Early graduation from child restraints to a seat belt has been reported to be associated with improper fit which can result in “jackknife” or “seat-belt syndrome” which are associated increased injury and mortality. Injury to the spine, abdomen, and head can occur from the passenger sliding beneath or bending around the seat belt (Durbin et al., 2003; Corden, 2005; Winston et al., 2004; Arbogast et al., 2002). Current AAP and NHTSA recommendations include use of booster seats for improved belt positioning until the child is of sufficient size to fit properly restrained in a seat belt (NHTSA, n.d.-a; Durbin & American Academy of Pediatrics, 2011). The findings of this study support the recommendation of the NHTSA and AAP for seating children under 12 years of age in the rear seat as they were less likely to be injured when rear-seated (NHTSA, n.d.-a; Durbin & American Academy of Pediatrics, 2011).

This study had limitations. It is possible that crashes not reported to the DMV and that did not result in injury being treated in an in-state hospital to have been missed. It is also possible to miss cases where injuries failed to match to a DMV crash report or if medical treatment was not sought or was sought out of the state. Other limitations which may hinder linkage include – crashes for which no prehospital crash report was made, reporting errors by public safety personnel, data transcribers or hospitals. These limitations are reflected in the specificity, and any errors in identifiable fields used for linkage may result in cases going unlinked. Failure to link could have underestimated the incidence of injury in this study population, but crashes that linked had high fidelity. Vehicles for hire have been shown to have lower overall restraint use and higher injury rates among children compared to private vehicles (Prince et al., 2019). This analysis did not differentiate between privately-owned vehicles and vehicles for hire such as taxi cabs, Uber, Lyft, and other ride-share modes of transit. Child and driver restraint status were assessed as a dichotomous variable. In our study, misclassification of being restrained could underestimate the protectiveness of being restrained if a driver falsely represented restraint status to law enforcement. Although it has been questioned whether driver’s falsely report being restrained to avoid a seatbelt fine, previous reports that used trained investigators, interviews of subjects and medical autopsy concluded that estimates of seat belt effects based on police-reported belt use, was not a significant source of bias in serious crashes involving a death (Cummings, 2002). This study did not assess fit of child restraint (Durbin et al., 2003) appropriateness of age for the child restraint or proper installation. Airbag deployment was used as a proxy variable for crash severity. Misclassification could have occurred if more airbags actually deployed than were captured in the data. This could lead to a potential underestimate of crash severity. This has been added to study limitations (Braver et al., 2010).

There are other factors known to contribute to increased child injury that were beyond the scope of this study (Oh et al., 2017; Huang et al., 2016; Pressley et al., 2007).

## Conclusions

CODES data is a powerful database containing hospital data merged with DMV vehicle, driver, occupant and crash characteristics for both nonfatal and fatal MV crashes. Use of this database facilitated our examination of crash injuries sustained in front vs. rear-seated, restrained and unrestrained, children aged 0–12 years and allowed us to investigate, in child occupants, reports that improvements in front seat safety have made this seating position less dangerous. Such reports have been used to question whether current recommendations that child passengers be rear-seated are dated and no longer reflective of best practices for the transport of children. The findings of this study comparing seating positions, restraint use and injury in NYS children aged 0–12 years involved in nonfatal as well as fatal MV crashes continues to lend support to the recommendations of AAP and NHTSA that children be restrained and rear seated. This study suggests that child safety legislation requiring children to be properly restrained in the rear seat continues to be a current best practice for the transport of children under age 12 years.

## Data Availability

Data will not be shared due to restrictions in data use agreements.
